# Surgical interventions to treat humerus shaft fractures: A network meta-analysis of randomized controlled trials

**DOI:** 10.1371/journal.pone.0173634

**Published:** 2017-03-23

**Authors:** Jia-Guo Zhao, Jia Wang, Xiao-Hui Meng, Xian-Tie Zeng, Shi-Lian Kan

**Affiliations:** 1 Departments of Orthopaedic Surgery, Clinical College of Orthopaedic Surgery, Tianjin Medical University, Tianjin, China; 2 Department of Orthopaedic Surgery, Tianjin Hospital, Tianjin, China; 3 Department of Orthopaedic Surgery, Yixing Traditional Chinese Medicine Hospital, Yixing, Jiangsu Province, China; University of Umeå, SWEDEN

## Abstract

**Background:**

There are three main surgical techniques to treat humeral shaft fractures: open reduction and plate fixation (ORPF), intramedullary nail (IMN) fixation, and minimally invasive percutaneous osteosynthesis (MIPO). We performed a network meta-analysis to compare three surgical procedures, including ORPF, IMN fixation, and MIPO, to provide the optimum treatment for humerus shaft fractures.

**Methods:**

MEDLINE, EMBASE, Cochrane Bone, Joint and Muscle Trauma Group Specialised Register, and Cochrane library were researched for reports published up to May 2016. We only included randomized controlled trials (RCTs) comparing two or more of the three surgical procedures, including the ORPF, IMN, and MIPO techniques, for humeral shaft fractures in adults. The methodological quality was evaluated based on the Cochrane risk of bias tool. We used WinBUGS1.4 to conduct this Bayesian network meta-analysis. We used the odd ratios (ORs) with 95% confidence intervals (CIs) to calculate the dichotomous outcomes and analyzed the percentages of the surface under the cumulative ranking curve.

**Results:**

Seventeen eligible publications reporting 16 RCTs were included in this study. Eight hundred and thirty-two participants were randomized to receive one of three surgical procedures. The results showed that shoulder impingement occurred more commonly in the IMN group than with either ORPF (OR, 0.13; 95% CI, 0.03–0.37) or MIPO fixation (OR, 0.08; 95% CI, 0.00–0.69). Iatrogenic radial nerve injury occurred more commonly in the ORPF group than in the MIPO group (OR, 11.09; 95% CI, 1.80–124.20). There were no significant differences among the three procedures in nonunion, delayed union, and infection.

**Conclusion:**

Compared with IMN and ORPF, MIPO technique is the preferred treatment method for humeral shaft fractures.

## Introduction

Fractures of the humerus shaft are relatively common, with an annual incidence rate varying from 12.0 and 23.4 fractures per 100,000 people and an increasing incidence with age.[1] Most fractures of the humeral shaft can be managed conservatively.[2] The indications for operative treatment include open fracture, pathological fracture, polytrauma, fracture with radial nerve or vascular injury, and failed non-surgical treatment leading to delayed or nonunion.[2, 3]

Plate and intramedullary nail (IMN) fixation are two traditional methods of fixation for the management of humeral shaft fractures. Open reduction and plate fixation (ORPF) allows direct visualization and anatomic reduction but also has potential disadvantages, such as radial nerve injury, and the risk of nonunion and deep infection resulting from extensive soft-tissue stripping.[4] Locked IMN is also the commonly used method of fixation for humeral shaft fracture. Theoretically locked IMNs are load-sharing devices that have less stress shielding, minimize the disruption of fracture biology, and allow the preservation of the periosteal blood supply.[5] However, the shoulder complications caused by IMN, such as shoulder impingement, cannot be neglected.[6, 7] Recently, the minimally invasive percutaneous osteosynthesis (MIPO) technique has been advocated to treat humeral shaft fractures.[8, 9] This technique minimizes the disruption of the fracture site and limits soft-tissue stripping compared with conventional open reduction and internal fixation.[4, 10]

Several systematic reviews or meta-analyses were performed to compare different internal fixation techniques for the surgical treatment of humeral shaft fracture.[11–13] However, these studies were inconclusive. In addition, traditional meta-analyses only directly compared two different interventions. Network meta-analysis can be used to pool evidence even if there are no head-to-head comparisons.[14, 15] In the current study, we performed a Bayesian network meta-analysis to compare three commonly used surgical procedures, including ORPF, MIPO, and IMN, to provide the optimum treatment method for humerus shaft fractures.

## Methods

We prospectively registered the protocol of this meta-analysis on the PROSPERP international prospective register of systematic reviews (CRD42016046918). We performed this systematic review and network meta-analysis based on “Preferred Reporting Items for Systematic Reviews and Meta-Analyses (PRISMA)” (S1 Checklist).[16]

### Search strategy

We used the search strategies described previously in the published protocol,[17] which were developed using the Cochrane Bone, Joint and Muscle Trauma Group. MEDLINE, EMBASE, Cochrane Bone, Joint and Muscle Trauma Group Specialised Register, and Cochrane library were researched for reports published up to May 2016. The following search words were used: “humeral fractures”, “humor* and fracture*”, and “shaft or midshaft or diaphys*”. We did not limit the publication status or language.

### Selection criteria

We selected trials based on following inclusion criteria: 1) randomized controlled trials; 2) a target population of adults over 16-year old with humeral shaft fractures; 3) trials comparing two or more of the three surgical procedures, including the ORPF, IMN, and MIPO technique (such as ORPF versus IMN). Exclusion criteria included the following conditions: 1) non- randomized controlled trials; 2) trials that enrolled children with humeral shaft fractures; 3) trials that enrolled adults with pathological or periprosthetic fractures; 4) trials only containing one or none of the three treatments (such as ORPF versus conservative treatment); 5) patients with nonunion of humeral shaft fractures following conservative or operative treatment. If a decision cannot be reached, differences were resolved by consultation with a third author.

### Study selection and data extraction

Two independent reviewers first screened the study titles and abstracts for eligibility. The full-text of the trials potentially meeting the eligibility criteria were reviewed to decide the final inclusion. Two investigators independently extracted information, including the lead author, publication year, randomization methods, participant number, patient characteristics (number, age and gender), follow-up time, loss to follow up, and all outcome measures.

### Assessment of methodological quality

The risk of bias tool of Cochrane collaboration was used to evaluate the methodological quality by two independent reviewers.[18] We assessed the items, including the random method, allocation concealment, blinding, incomplete outcome data, selective reporting, and any other possible bias such as the baseline between different groups. The determination of the level of evidence was assessed according to the Oxford Centre for Evidence-based Medicine Levels of Evidence.

### Data analysis

According to the statistical method described by Chaimani et al.[19], this Bayesian network meta-analysis was conducted using WinBUGS1.4. We used the odd ratios (ORs) with 95% confidence intervals (CIs) to calculate the dichotomous outcomes and standardized mean difference (SMD) with 95% CIs to calculate continuous outcomes. The ranking of all of the evaluated surgical methods for the outcome measures could be provided in this Bayesian network meta-analysis.[20] We calculated the percentages of the surface under the cumulative ranking curve (SUCRA). A higher SUCRA percentage means better results for the respective intervention.[20] We used the funnel plot to detect the presence of small-study effects. The inconsistencies of any available direct and indirect estimates were assessed by comparing statistics for the deviance information criterion. The graphical tools in STATA12 were used to show the network diagram and funnel plot.

## Results

### Search results

**Fig 1:**
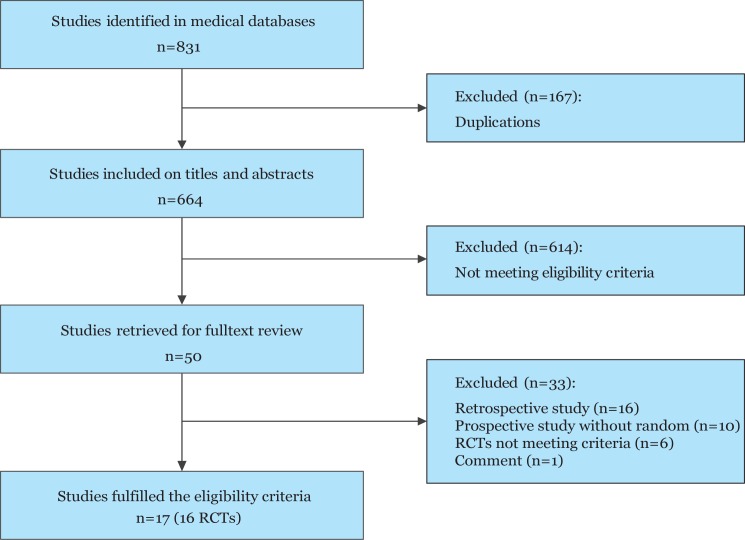
Flow diagram depicting the study selection for inclusion in the meta-analysis. RCT = randomized controlled trial.

**Fig 2:**
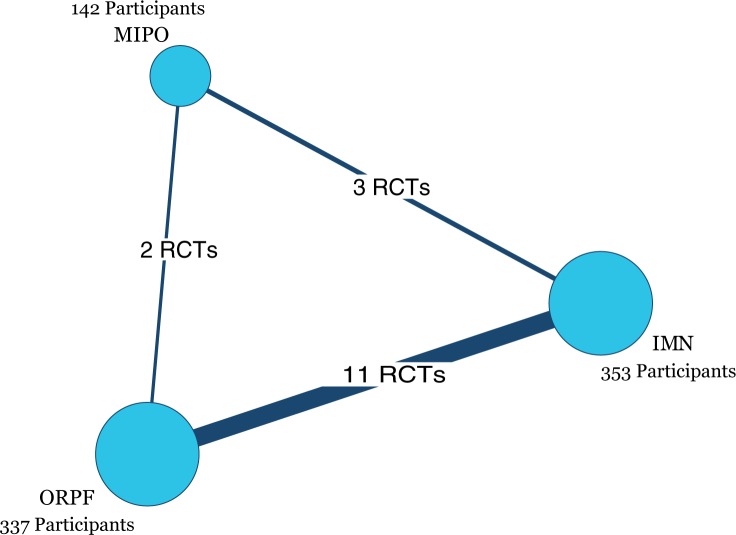
Network of the comparisons for the Bayesian network meta-analysis. IMN = intramedullary nailing; MIPO = minimally invasive percutaneous osteosynthesis; ORPF = open reduction and plate fixation; RCT = randomized controlled trial.

Eight hundred sixty-one potentially relevant records from database searches were identified (Fig 1). Of these references, we read 50 full-text potential publications. Seventeen eligible publications [21–37] reporting 16 RCTs were included in this Bayesian network meta-analysis. All of the RCTs were published in English except for one in Czech.[23] Eight hundred thirty-two participants were randomized to receive one of three surgical procedures. Individual sample sizes ranged from 28 to 89 participants. Fig 2 presents three comparisons within the network and the number of RCTs for each comparison. The characteristics of all RCTs are summarized in Table 1.

**Table 1:** Characteristics of the 16 included studies.

Study (Year)	Design	Number of Patients	Mean age (Year)	Female Patients (%)	Intervention	Comparison	Follow up time (months)	Loss to follow up
Benegas 2014	RCT	40	41.7	14 (34%)	MIPO	IMN	12	2.4%
Bolano 1995	RCT	28	NA	NA	ORPF	IMN	NA	10.7%
Changulani 2007	RCT	47	37.0	8 (17%)	ORPF	IMN	14	4.3%
Chapman 2000	RCT	89	33.5	33 (37%)	ORPF	IMN	13	5.6%
Esmailiejah 2015	RCT	68	34.0	17 (25%)	ORPF	MIPO	NA	4.4%
Fan 2015	RCT	60	39.3	23(38%)	ORPF	IMN	12	0%
Kesemenli 2003	RCT	60	38.0	17 (28%)	ORPF	IMN	42	0%
Kim 2015	RCT	72	42.5	31 (43%)	ORPF	MIPO	15	5.6%
Li 2011	RCT	50	37.6	13 (26%)	ORPF	IMN	18	4%
Lian 2013	RCT	56	38.2	8 (15%)	MIPO	IMN	14.5	16%
McCormack 2000	RCT	44	44.7	16 (36%)	ORPF	IMN	14.3	6.8%
Putti 2009	RCT	34	37.6	2 (5.9%)	ORPF	IMN	24	0%
Shah 2015	Quasi-RCT	40	NA	10 (25%)	ORPF	IMN	NA	0%
Singisetti 2010	Quasi- RCT	45	NA	8 (22%)	ORPF	IMN	12	20%
Smejkal 2014	RCT	49	51.3	22 (49%)	MIPO	IMN	NA	8%
Wali 2014	RCT	50	37.5	9 (18%)	ORPF	IMN	13	0%

Abbreviations: IMN = intramedullary nailing; MIPO = minimally invasive percutaneous osteosynthesis; ORPF = open reduction and plate fixation; RCT = randomized controlled trial.

### Methodological quality

**Fig 3:**
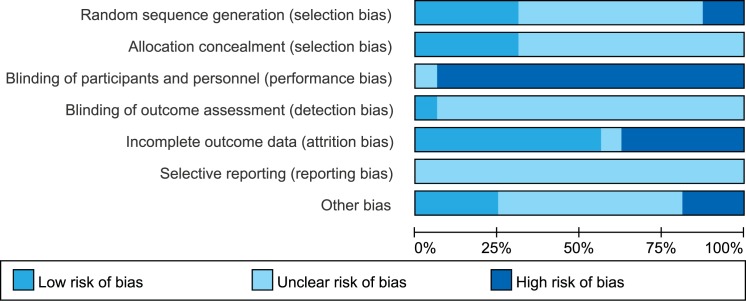
Risk of bias graph. Each risk of bias item was presented as a percentage across all of the included studies.

Although all of the studies reported randomization, only five trials[23, 28, 29, 32, 36] described an adequate randomization procedure, and five trials[23, 27, 29, 33, 34] reported adequate concealment. Two trials were considered as quasi-RCTs.[24, 37] Blinding was not possible for the participants and clinicians because of the nature of the surgical interventions. The risk of bias of the included RCTs is shown in Fig 3. Based on the Oxford Centre for Evidence-based Medicine Levels of Evidence, all of the trials were assessed as Level II evidence.

### Results of network meta-analysis

#### Nonunion

**Fig 4:**
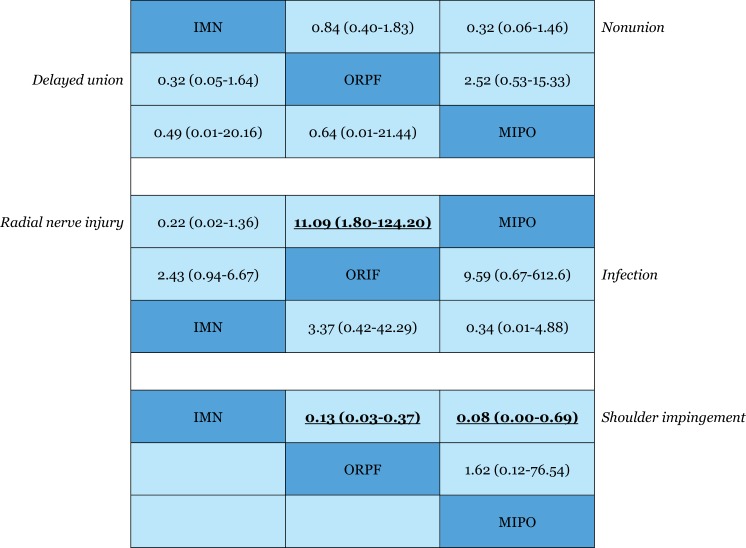
Odd ratios with 95% confidence intervals for the outcome measures. The estimate is in the cell in common between the column-defining treatment and row-defining treatment. Significant results are in bold and are underlined. IMN = intramedullary nailing; MIPO = minimally invasive percutaneous osteosynthesis; ORPF = open reduction and plate fixation.

**Fig 5:**
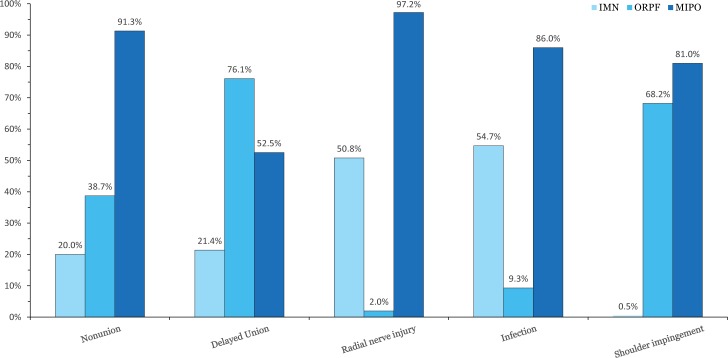
Surface under the cumulative ranking curves for the outcome measures. IMN = intramedullary nailing; MIPO = minimally invasive percutaneous osteosynthesis; ORPF = open reduction and plate fixation.

All of the included RCTs reported the rate of nonunion. The results of this network meta-analysis suggested that there were no significant differences among the three procedures in nonunion. The OR values and 95% CIs are summarized in Fig 4. The SUCRA probabilities were 20.0%, 38.7%, and 91.3% for IMN, ORPF, and MIPO, respectively. In Fig 5, we summarized the SUCRA probability of nonunion for the three treatment methods.

#### Delayed union

Delayed union was reported in five included RCTs.[22, 23, 28, 29, 33] The result of the network meta-analysis suggested that there were no significant differences among the three procedures in delayed union. The OR values and 95% CIs are summarized in Fig 4. Furthermore, the SUCRA probabilities were 21.4%, 76.1%, and 52.5% for IMN, ORPF, and MIPO, respectively (Fig 5).

#### Iatrogenic radial nerve injury

All of the included RCTs reported the rate of iatrogenic radial nerve injury. The pooled result showed a significantly higher occurrence of iatrogenic radial nerve injury in the ORPF group than in the MIPO group (OR, 11.09; 95% CI, 1.80–124.20) (Fig 4). There was no significant difference between MIPO and IMN in the iatrogenic radial nerve injury rate. The SUCRA probabilities were 50.8%, 2.0%, and 97.2% for IMN, ORPF, and MIPO, respectively (Fig 5).

#### Infection

We only pooled the data from the RCTs exclusively including closed fracture because the number of open fractures was imbalanced between the two controlled groups in several RCTs. Seven trials only included closed fractures and reported the rate of infection.[22, 23, 27, 28, 30, 32, 37] The network meta-analysis suggested that there were no significant differences among the three procedures in infection. The OR values and 95% CIs are summarized in Fig 4. The SUCRA probabilities were 54.7%, 9.3%, and 86.0% for IMN, ORPF, and MIPO, respectively (Fig 5).

#### Shoulder impingement

Ten included RCTs reported the rate of shoulder impingement.[22, 23, 25–27, 30, 33–35, 37] The pooled result showed a significantly higher occurrence of shoulder impingement in the IMN group than in either the ORPF (OR, 0.13; 95% CI, 0.03–0.37) or MIPO group (OR, 0.08; 95% CI, 0.00–0.69) (Fig 4). The SUCRA probabilities were 0.5%, 68.2%, and 81.0% for IMN, ORPF, and MIPO, respectively (Fig 5).

### Small-study effect and inconsistency test

**Fig 6:**
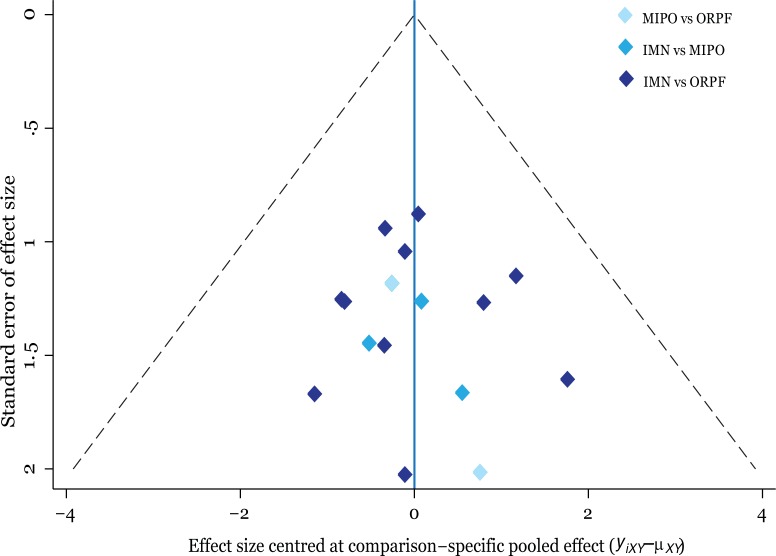
Funnel plot of the network meta-analysis. IMN = intramedullary nailing; MIPO = minimally invasive percutaneous osteosynthesis; ORPF = open reduction and plate fixation.

Fig 6 shows that the funnel plot is symmetrical, indicating there is no small-study effect in this network meta-analysis. The result of the inconsistency test between direct and indirect comparisons showed that the statistical inconsistency was generally low because the CI values included zero.

## Discussion

Conservative treatment for humeral shaft fractures can lead to serious complications such as nonunion and malunion.[4] Hence, surgical treatment plays an important role. The following are currently the three main surgical techniques to treat humeral shaft fractures: ORPF, IMN fixation, and the more recently described MIPO technique. Several systematic reviews regarding the management of humeral shaft fractures have been published.[11, 13, 38] Most of them have focused on the comparison between the plate and IMN fixation. We summarized the overlapping meta-analyses and found that IMN significantly increased the incidence of shoulder complications such as shoulder impingement.[7] In comparing three or more different treatments, the combination of the present evidence using traditional meta-analysis is impossible. Network meta-analysis using the Chaimani model is a well-confirmed approach because it compares 3 or more interventions for a clinical question.[15]

The MIPO technique has the biomechanical advantage for comminuted shaft fractures. Recently, the MIPO technique was also used in simple fractures,[27, 29, 32] allowing the theoretical benefits of less soft-tissue stripping and exposure. The MIPO technique using the relative stability principle in shaft fractures has gained popularity for its potential advantage in causing fewer soft-tissue complications.[8, 9, 39, 40] Open reduction and internal fixation through either an anterolateral or posterior approach needs significant soft-tissue and local vascularity disruption, which may lead to a decreased fracture healing potential and increase the incidence of iatrogenic radial nerve injury and deep infection.[33] This meta-analysis showed that the MIPO technique had a significantly lower incidence rate of iatrogenic radial nerve injury than ORPF. Although there was no significant difference between MIPO and ORPF according to the OR values, the SUCRA percentage showed that MIPO had a lower probability of nonunion and infection than ORPF.

IMN fixation is a minimally invasive technique that avoids the problems encountered with ORPF, with less disruption for the blood supply of the fracture site during the surgery.[11] However, IMN fixation has been shown to cause a higher risk of shoulder complications than plates such as shoulder impingement.[7, 17] This network meta-analysis suggested that there were no significant differences between MIPO and IMN based on OR values in nonunion and iatrogenic radial nerve injury. However, either MIPO or ORPF significantly decreased the postoperative rate of shoulder impingement compared with IMN.

Our study has several strengths. First, this study can be considered the first network meta-analysis of a randomized trial that evaluates the surgical procedures for humeral shaft fractures. Although several meta-analyses regarding this title have been published, [12, 13, 38] none are network meta-analyses. Second, a major strength of present study is that all of the included studies used a randomized controlled design, which increases the comparability between the two groups and reduces the probability of selection bias. Our meta-analysis exclusively included 16 randomized controlled trials, and finally we obtained evidence at a high level. Third, search strategies were developed using the Cochrane Bone, Joint and Muscle Trauma Group. The current meta-analysis included more RCTs through a more extensive search.

The main limitation of the current meta-analysis was that there were insufficient studies to permit the evaluation of shoulder function. Although several trials reported the shoulder functional scores, some of them did not report the complete data of 95% CIs or standard deviation. Unlike the postoperative complications, shoulder scores varies with different follow-up time. It is not appropriately to pool them in a network meta-analysis because of the presence of inconsistency. In addition, there were two types of plates in the trials including the dynamic compression plate and locking compression plate. We did not divide them into two groups because of the limited number of included RCTs.

## Conclusion

In summary, compared with IMN, either ORPF or MIPO significantly decreased the risk of shoulder impingement. Furthermore, the pooled results showed a significantly higher occurrence of iatrogenic radial nerve injury in the ORPF group than in the MIPO group. There were no significant differences among the three procedures in nonunion, delayed union, and infection. Hence, we concluded that the MIPO technique is the preferred treatment method for humeral shaft fractures.

## Supporting information

S1 PRISMA Checklist Preferred Reporting Items for Systematic Reviews and Meta-Analyses (PRISMA). (DOC)
